# A network-based approach for isolating the chronic inflammation gene signatures underlying complex diseases towards finding new treatment opportunities

**DOI:** 10.3389/fphar.2022.995459

**Published:** 2022-10-12

**Authors:** Stephanie L. Hickey, Alexander McKim, Christopher A. Mancuso, Arjun Krishnan

**Affiliations:** ^1^ Department of Biochemistry and Molecular Biology, Michigan State University, East Lansing, MI, United States; ^2^ Department of Computational Mathematics, Science and Engineering, Michigan State University, East Lansing, MI, United States; ^3^ Genetics and Genome Sciences Program, Michigan State University, East Lansing, MI, United States; ^4^ Department of Biostatistics and Informatics, Colorado School of Public Health, University of Colorado-Denver Anschutz Medical Campus, Aurora, CO, United States; ^5^ Department of Biomedical Informatics, University of Colorado Anschutz Medical Campus, Aurora, CO, United States

**Keywords:** complex disease, inflammation, endophenotype, drug repurposing, network analysis, functional modules, disease modules

## Abstract

Complex diseases are associated with a wide range of cellular, physiological, and clinical phenotypes. To advance our understanding of disease mechanisms and our ability to treat these diseases, it is critical to delineate the molecular basis and therapeutic avenues of specific disease phenotypes, especially those that are associated with multiple diseases. Inflammatory processes constitute one such prominent phenotype, being involved in a wide range of health problems including ischemic heart disease, stroke, cancer, diabetes mellitus, chronic kidney disease, non-alcoholic fatty liver disease, and autoimmune and neurodegenerative conditions. While hundreds of genes might play a role in the etiology of each of these diseases, isolating the genes involved in the specific phenotype (e.g., inflammation “component”) could help us understand the genes and pathways underlying this phenotype across diseases and predict potential drugs to target the phenotype. Here, we present a computational approach that integrates gene interaction networks, disease-/trait-gene associations, and drug-target information to accomplish this goal. We apply this approach to isolate gene signatures of complex diseases that correspond to chronic inflammation and use SAveRUNNER to prioritize drugs to reveal new therapeutic opportunities.

## 1 Introduction

Acute inflammation is an organism’s healthy response to invasion by pathogens or to cellular damage caused by injury (Rock and Kono 2008). Systemic chronic inflammation (CI) occurs when these inflammatory responses do not resolve, resulting in persistent, low-grade immune activation that causes collateral damage to the affected tissue over time (Furman et al., 2019). While the direct connection of CI to autoimmune diseases has been well known for some time, only recently has the medical community uncovered the prevalence of CI in a multitude of complex diseases and disorders (Furman et al., 2019; Vos et al., 2020). Therefore, it is imperative to better understand the different molecular mechanisms of CI manifestation across diseases.

Network-based methods are powerful collection of tools for both elucidating specific pathways and processes that may underlie a complex phenotype (Ghiassian et al., 2015; Leiserson et al., 2015; Ghiassian et al., 2016) and for drug repurposing (Chen et al., 2015; Cheng et al., 2018; Fiscon and Paci 2021). For instance, HotNet2 is a pan-cancer network analysis tool that identifes active network modules in a genome-wide molecular network by guiding the module detection algorithm with thousands of genes scored by how prevalent they are across 12 cancers in TCGA (Leiserson et al., 2015). HotNet2 is then able to determine if any module is enriched for a given cancer type, pathway, or process. In a similar vein, another approach, DIAMOnD, starts with a genome-wide network, and then creates a disease-specific network using an expanded set of known disease-gene annotations (Ghiassian et al., 2015). This disease-specific network is then analyzed and compared to other disease-specific networks generated using the same technique. Both approaches find regions of a genome-wide network that are enriched for disease-related genes.

Inflammation is an example of an endophenotype, or intermediate phenotype, of a complex disease. Ghiassian *et al.* studied endophenotype network models by starting with a genome-wide network and constructing modules for sets of seed genes related to three endophenotypes: inflammation, thrombosis, and fibrosis (Ghiassian et al., 2016). The authors showed that the network modules derived from the three endophenotypes have strong overlap in the network and that these modules are enriched for genes differentially expressed in various complex diseases. While the above methods provide invaluable insight in disease mechanisms using a disease-focused and a phenotype-focused approach, respectively, they raise the critical question of identifying phenotypic signatures specific to individual diseases. For instance, can we identify the CI-signature that is specific to a given disease and use that to find avenues for therapeutic intervention?

**FIGURE 1:**
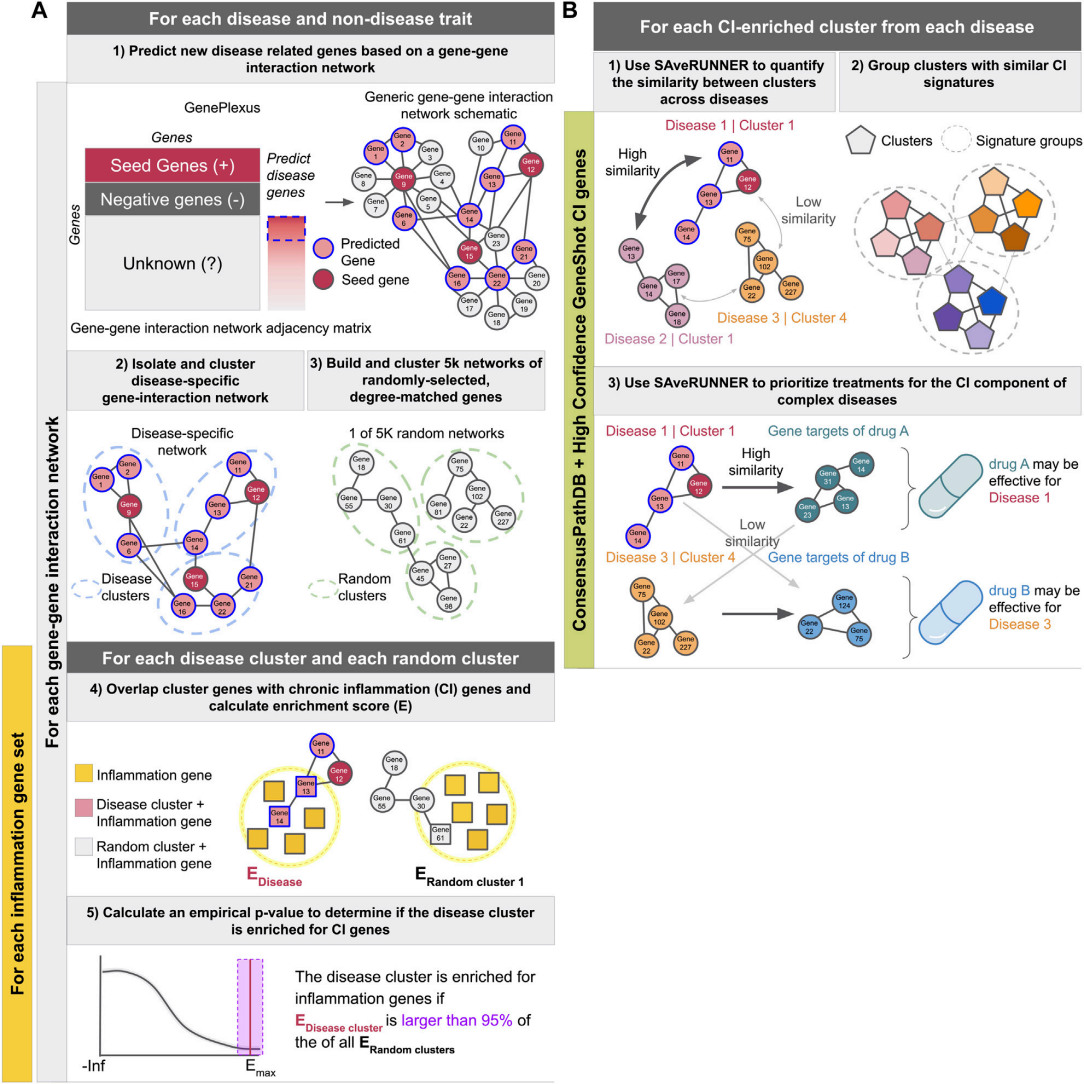
Schematics describing the experimental pipeline. **(A)** Describes predicting new disease related genes (step 1), clustering the disease-specific interaction network (step 2) as well as 5,000 networks made from randomly-selected degree-matched genes for each disease (step3), identifying CI-enriched clusters (steps 4 and 5), and calculating the proportion of diseases with at least one CI-enriched cluster. These steps were performed for each gene-gene interaction network in combination with each inflammation gene set described in the methods. **(B)** Describes using SAveRUNNER to find groups of CI-enriched clusters from all diseases with similar CI-signatures (steps 1 and 2), and prioritize treatments for the CI component of complex diseases (step 3). Using ConsensusPathDB with the high-confidence GeneShot derived CI gene set resulted in the highest proportion of autoimmune diseases and the lowest proportion of non-disease traits with at least one CI-enriched cluster. Therefore, steps one to three were performed with clusters from that network-CI gene set combination only.

In this work, we address this question using a network-based approach. We first generate a network consisting of only genes associated with a single disease (Figure 1A, steps 1–2) Here, like in DIAMOnD (Ghiassian et al., 2015), we expand our original disease-gene annotations to build more robust networks and glean insight into unstudied genes. We use a network-based supervised machine learning model, shown to systematically outperform label propagation methods like DIAMOnD, to expand our gene sets (Liu et al., 2020). We then cluster the disease-specific network, find clusters that are significantly enriched for known CI genes, and compare these CI signatures across diseases (Figures 1A,B steps 12). We then use the SAveRUNNER (Fiscon and Paci 2021) method on these enriched clusters to predict drugs that might help treat the CI-component specific to a given disease (Figure 1B, step 3).

## 2 Methods

### 2.1 Disease selection and disease-associated seed genes

#### 2.1.1 Complex and autoimmune diseases

We searched the literature (Furman et al., 2019; Dregan et al., 2014; Armstrong et al., 2013; Yashiro 2014; Chou et al., 2016; Autoimmune Diseases: Causes, 2022) and curated examples of 17 complex diseases associated with chronic inflammation (CI) and nine common autoimmune diseases. Some of these diseases are quite broad (i.e “Malignant neoplasm of lung”), and to add more narrowly defined diseases to our list, we used the Human Disease Ontology (Schriml et al., 2019) to identify child terms of these diseases. The chosen diseases were not meant to be comprehensive, but examples of autoimmune diseases and complex diseases thought to have immune components. We then identified genes annotated to each disease by the DisGeNet database, which is a database that stores a collection of disease-gene annotations from expert curated repositories, GWAS catalogs, animal models and the scientific literature (Piñero et al., 2020). To ensure that our disease gene sets were largely non-overlapping, we created a network such that nodes were diseases, and an edge was created between two diseases if the two gene sets had ≥0.6 overlap ( |A∩B|/min⁡⁡(|A|, |B|)) . We then chose the most representative disease from each connected component. This resulted in 10 autoimmune diseases and 37 complex diseases (Supplementary Table S1).

#### 2.1.2 Non-disease traits

Two lab members manually curated 113 non-disease-traits that are unlikely to be associated with CI (i.e. handedness, coffee intake, and average household income) from the list of traits with GWAS results from the UK Biobank (Sudlow et al., 2015) to be used as negative controls. Based on GWAS summary statistics from the Neale group (Abbot et al., 2021), we used Pascal (Lamparter et al., 2016) (upstream and downstream windows of 50 KB with the sum-of-chi-squared statistics method; only autosomal variants) to associate genes with the non-disease traits. Genes with p<0.001 were included as seed genes for that trait.

### 2.2 GenePlexus

To predict new genes associated with a set of input seed genes, we used GenePlexus, a tool that builds an L2-regularized logistic regression model using features from a gene interaction network (Liu et al., 2020). As input features, we used the adjacency matrices from STRING, STRING with only experimentally derived edges (STRING-EXP) (Szklarczyk et al., 2017), BioGRID (Stark et al., 2006), and ConsensusPathDB (Kamburov et al., 2013). For predicting disease genes, positive examples were disease/trait seed genes and negative example genes were generated by: (i) finding the union of all genes annotated to all diseases in DisGeNET (Piñero et al., 2020), (ii) removing genes annotated to the given seed genes, and (iii) removing genes annotated to any disease in the collection that significantly overlapped with the given seed genes ( p<0.05 based on the one-sided Fisher’s exact test) (Liu et al., 2020). We tested the performance of the above features for predicting new genes associated with our diseases and traits of interest using three-fold cross validation and only included diseases in subsequent analyses if the diseases/traits had ≥15 associated genes and median $\log_{2}\left(auPRC/prior\right)\geq 1$ (i.e. the area under the precision-recall curve ‘auPRC’ is at least twice as much as expected by random chance ‘prior’ (Liu et al., 2020)). See Figure 1A, step 1 and Supplementary Table S1.

### 2.3 Identifying clusters of interacting genes within a disease-specific network

One list of disease-associated genes was formed for each of the four biological networks used as features in GenePlexus. Specifically, we added genes with a GenePlexus prediction probability of ≥0.80 on the network of interest to the original disease or trait seed gene list to create our final set of associated genes for each disease or trait for that network. We formed disease-/trait-specific networks by subsetting a given network to include only the disease-/trait-associated genes and any edges directly connecting those genes (Figure 1A, step 2). We tested five prediction-network—cluster-network combinations: Genes predicted on each of the four networks were clustered on the same network. Genes predicted on STRING were also clustered on both STRING and STRING-EXP to test if using the full network for novel gene prediction but only experimentally derived gene-gene associations for clustering would improve performance. We then used the Leiden algorithm (Traag et al., 2019) to partition the disease-/trait-specific networks into clusters (Figure 1A, step 2). Specifically, we used the *leiden_find_partition* function from the leidenbase R package (v 0.1.3) (https://github.com/cole-trapnell-lab/leidenbase) with 100 iterations and *ModularityVertexPartition* as the partition type. We retained clusters containing ≥5 genes.

### 2.4 Cluster GOBP enrichment analysis

We used the R package topGO with the “weight01” algorithm and Fisher testing (Alexa and Rahnenfuhrer 2022) (v 2.44.0) to find enrichment of genes annotated to GO biological processes (min size = 5, max size = 100) among disease gene clusters. The annotations were taken from the Genome wide annotation for Human bioconductor annotation package, org.Hs.eg.db (Carlson 2019) (v 3.13.0). The background gene set included all human genes present in the network of interest. This was performed for every prediction/clustering method combination.

### 2.5 Isolating CI-associated disease clusters

#### 2.5.1 Defining CI-associated genes

We tested several different sets of chronic inflammation associated genes for this study including the GO biological process (GOBP) terms GO:0002544 (“chronic inflammatory response”) and GO:0006954 (“inflammatory response”). These were collected from the *Genome wide annotation for Human* bioconductor annotation package, org. Hs.eg.db (Carlson 2019) (v 3.13.0) with and without propagation of gene-term relationships from the descendent terms (org.Hs.egGO2ALLEGS and org.Hs.egGO2EG, respectively). GO:0006954 was also filtered to retain gene-term relationships inferred from experiments (evidence codes EXP, IDA, IPI, IMP, IGI, IEP, HTP, HDA, HMP, HGI, and HEP). As GO:0002544 without propagation contained <15 genes, this list was ultimately not included in the study. We also identified genes associated with chronic inflammation using Geneshot which, given the search term “chronic inflammation”, searches Pubmed using manually collected GeneRif gene-term associations to return a ranked list containing genes that have been previously published in association with the search term (Lachmann et al., 2019). We tested both the entire Geneshot generated list, and the subset of genes with >10 associated publications (“High-confidence GeneShot”). As with the disease genes, we predicted additional chronic-inflammation-associated genes using GenePlexus with features from each network. Negative examples for GenePlexus were derived from non-overlapping GOBP terms. We added genes with a prediction probability of ≥0.80 to the seed gene list to create our final sets of CI-associated genes.

#### 2.5.2 Creating random traits

After running GenePlexus to predict new genes for each trait, the gene lists for each trait were used to generate 5,000 random gene lists that have matching node degree distributions to the original traits (Figure 1A, step 3). That is, a random gene list was generated for a given trait by replacing each of its genes in the network of interest with a (randomly chosen) gene that has the same node degree, or a gene that has a close node degree if there are a small number of genes with the exact node degree (Leiserson et al., 2015; Fiscon and Paci 2021). We clustered the random traits as described in Section 2.3. Only clusters with at least five genes were included. Real traits with no corresponding permuted traits with clusters containing at least five genes were excluded from the analysis.

#### 2.5.3 Finding CI-gene enriched disease clusters

For each prediction-network—cluster-network pair and each CI gene list expanded on the prediction network of interest, for each disease and random trait cluster containing ≥5 genes, we calculated an enrichment score:

$$E=\log_{2}\left(\frac{\left(CG\cap CI\right)/CG}{CI/background}\right)$$

where CG are the genes in a disease cluster, CI are the CI genes, and background is all the genes present in the clustering network (Figure 1A, step 4). For each real disease or trait cluster, we used the matching random trait clusters to calculate a *p*-value:

$$p=\frac{\sum_{i=1}^{n}x_{i}}{n+1}$$

where n is the number of random trait clusters from all 5k matching random traits, and

$$x_{i}=\left\{ \begin{array}{c} 1,\;E_{random\;cluster\;i}\geq E_{disease\;cluster}\;\\ {0,\;E}_{random\;cluster\;i}<E_{disease\;cluster\;} \end{array}\right.$$

We corrected for multiple comparisons across clusters within a disease using the Benjamini–Hochberg procedure (Benjamini and Yosef 1995) (Figure 1A, step 5). Clusters with an FDR<0.05 and E>0 were considered chronic-inflammation-associated disease clusters and were deemed to represent the ‘CI signature’ of the disease.

#### 2.5.4 Identifying the optimal prediction-network/cluster-network/CI gene source combination

We chose the network/inflammation gene set combination that resulted in the highest proportion of autoimmune diseases and lowest proportion of non-disease traits with at least one CI-enriched cluster of any network/CI-gene set combination, ConsensusPathDB and the high-confidence Geneshot generated list.

#### 2.5.5 Comparing CI-signatures across diseases

For CI-enriched clusters identified using ConsensusPathDB and the high-confidence Geneshot CI genes, we used the SAveRUNNER R package to quantify the similarity between each pair of CI-enriched clusters using ConsensusPathDB as the base network (Fiscon and Paci 2021) (Figure 1B, step 1). For each pair, SAveRUNNER computes the average shortest path between each gene in cluster A and the closest gene in cluster B and uses this value to calculate an adjusted similarity score. Then, a *p*-value is estimated based on a null distribution of adjusted similarity scores between randomly generated clusters with the same node degree distributions as clusters A and B. Because the similarity scores and *p*-values are not symmetric (i.e., A→B≠B→A ) we used Stouffer’s method to combine *p*-values for the same pair of clusters and averaged the adjusted similarities. We then used the Leiden algorithm as described in Section 2.3 to group related clusters (Figure 1B, step 2). For each group, we took the union of the genes belonging to the resident CI-enriched clusters. Using genes unique to each group, with all the ConsensusPathDB genes as background, we used TopGO as in Section 2.4 to identify enriched GOBPs.

### 2.6 Predicting novel treatment opportunities

#### 2.6.1 Identifying expert-curated drug-target associations

The known drug-gene interactions used in this study are the subset of the interactions present in the DrugCentral database (Avram et al., 2021) that are also among the expert curated interactions in the Drug-Gene Interaction database (DGIdb) (Freshour et al., 2021). Specifically, we used the DGIdb API to retrieve only drug-gene interactions that were marked “*Expert curated*” (based on the source trust levels endpoint). Intersecting these interactions with those in DrugCentral (through a list of drug synonyms from DrugCentral) resulted in the final list of expert-curated drug-gene pairs.

#### 2.6.2 Treatment prediction and scoring

We predicted treatment opportunities for the inflammatory component of complex diseases by using the SAveRUNNER R package (Fiscon and Paci 2021) (Figure 1B, step 3). SAveRUNNER builds a bipartite drug-disease network by utilizing the previously determined expert-curated drug targets, the CI-associated cluster disease genes, and the ConsensusPathDB network as a human interactome. Network similarity scores returned by SAveRUNNER represent the proximity between disease and drug modules, where a high similarity score means that the disease and drug modules have high proximity in ConsensusPathDB. SAveRUNNER calculates a *p*-value where a significant value suggest that the disease genes and drug targets closer in the network than expected by chance (based on an empirical null distribution built using 200 pairs of randomly selected groups of genes with the same size and degree distribution of the original sets of disease genes and drug targets). Using the list of final predicted associations after normalization of network similarity, the *p*-values were corrected for multiple comparisons within each disease using the Benjamini–Hochberg procedure. Drugs were associated to diseases based on the disease cluster with the lowest FDR. Predicted treatments are disease-drug pairs with an FDR<0.01 .

#### 2.6.3 Evaluating SAveRUNNER prediction performance

We calculated $\log_{2}\left(auPRC/prior\right)$ by ranking disease-drug pairs by $-\log_{10}\left(SAveRUNNER\;FDR\right)$ and using either previously indicated drug-disease pairs (both approved and off-label) or drug-disease pairs tested in a clinical trial as positive labels. Approved and off-label drug-disease pairs were collected from DrugCental (Avram et al., 2021). Only drugs with expert curated target genes were included (see Section 2.6.1). The Unified Medical Language System (UMLS) Concept Unique Identifiers (CUI) were limited to diseases (T047) and neoplastic processes (T191), and our diseases were matched to diseases in DrugCentral using UMLS CUIs. Drug-disease pairs tested in a clinical trial were collected from the database for Aggregate Analysis of Clinical Trials (AACT) (AACT Database, 2022). AACT reports the Medical Subject Headings (MeSH) vocabulary names for diseases. We used disease vocabulary mapping provided by DisGeNET to translate UMLS CUIs for our diseases to MeSH vocabulary names, further restricted to only those that were present in AACT. We filtered AACT for trials with “Active, not recruiting”, “Enrolling by invitation”, “Recruiting”, or “Completed” status.

#### 2.6.4 Enrichment of predicted drug-disease pairs among previously indicated drug-disease pairs

To test for an enrichment of predicted drug-disease pairs among previously indicated drug-disease pairs for each disease, we tallied the total number of unique drugs previously indicated to any disease, the number of those drugs indicated to the disease of interest, the number of drugs predicted to treat the disease by our method, and the number of drugs predicted to treat the disease by our method that were also previously indicated for that disease. We calculated a *p*-value using a one tailed Fisher’s exact test and corrected for multiple comparisons within each disease across drugs using the Benjamini–Hochberg procedure.

#### 2.6.5 Enrichment of anti-inflammatory drugs and immunosuppressants among predicted treatments

We searched the DrugBank database for the ATC codes for anti-inflammatory drugs and immunosuppressants including Immunosuppressants (L04), Corticosteroids for systemic use (H02), Anti-inflammatory and antirheumatic products (M01), and Antihistamines (R06) (Wishart et al., 2018). We used these codes to pull all the drugs in these categories from our expert curated drug to target gene database. For each disease we ranked predicted drugs by ${-\log}_{10}\left(SAveRUNNER\;FDR\right)$ and used the fgsea R package (v 1.20.0) to perform gene set enrichment analysis for drugs belonging to each of the four classes (Subramanian et al., 2005; Korotkevich et al., 2021).

## 3 Results

### 3.1 Expanding lists of disease-related genes and identifying disease-specific gene subnetworks

Our first goal was to establish a comprehensive list of genes associated with the complex diseases of interest and resolve the genes linked to each disease into subsets of tightly connected genes in an underlying molecular network. Towards this goal, we selected 37 complex diseases associated with underlying systemic inflammation (see *Methods*). To ensure that we correctly isolate chronic inflammation (CI) signatures, we devised a set of positive and negative controls. We selected 10 autoimmune disorders as positive controls because autoimmune disorders are characterized by CI and should have an easily identifiable CI gene signature. For negative controls, we selected 113 traits from UK Biobank (Sudlow et al., 2015) that are unlikely to be associated with CI (i.e. Right handedness, filtered coffee intake, and distance between home and workplace). Supplementary Table S2 contains the full list of diseases and traits used in this analysis along with their original associated genes.

While thousands of genes may play a role in the etiology of a chronic disease, it is unlikely that all of these genes have been cataloged in available databases such as DisGeNET or identified by GWAS. Hence, we expanded the lists of disease-or-trait-associated genes using GenePlexus (Liu et al., 2020) (Figure 1A, step 1). Briefly, GenePlexus performs supervised machine learning using network-based features to predict novel genes related to a set of input seed genes. Here, we built one GenePlexus model per disease using disease-associated genes from DisGeNET or trait-associated genes from the UK Biobank GWAS as seed genes (positive examples). To test the robustness of this method for identifying CI enriched clusters, we tested four different biological interaction networks of varying sizes and edge densities—STRING, STRING with only experimentally derived edges (STRING-EXP) (Szklarczyk et al., 2017), BioGRID (Stark et al., 2006), and ConsensusPathDB (Kamburov et al., 2013) (Figure 1A, step 1, see Methods Section 2.2). Genes predicted by the GenePlexus model with a probability ≥0.80 were added to the seed gene list to create an expanded list of disease- or trait-associated genes.

**FIGURE 2:**
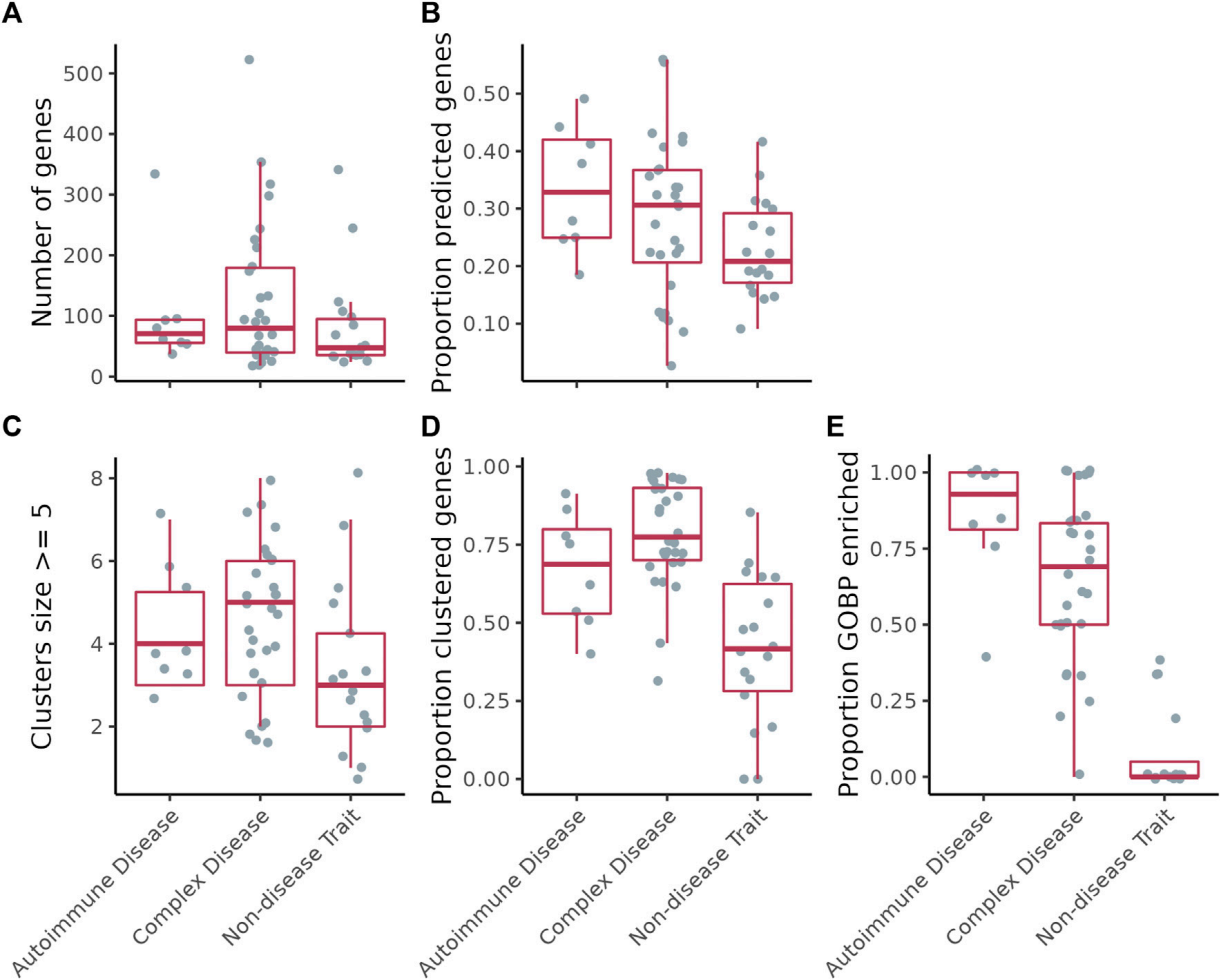
**(A)** Number of genes per disease/trait. **(B)** Proportion of the genes per disease/trait that were predicted by GenePlexus. **(C)** Number of clusters per disease/trait containing at least five genes. **(D)** Proportion of total genes assigned to a cluster containing at least five genes. **(E)** Proportion of clusters per disease/trait enriched with genes from at least one GO biological process.

Figure 2 shows results for ConsensusPathDB. The proportion of genes predicted by GenePlexus for the non-disease traits is lower than those for the autoimmune and complex diseases (Figure 2B). This observation indicates that genes associated with a specific autoimmune/complex disease tend to have more similar network neighborhoods than genes associated with non-disease traits. All disease-associated genes after GenePlexus prediction are listed in Supplementary Table S3.

Next, for each disease/trait, we clustered the expanded lists of genes based on their interactions in the gene-gene interaction network (Figure 1A step 2 and Figure 2C; Supplementary Table S3). On ConsensusPathDB, the complex diseases had the highest proportion of genes grouped into clusters of ≥5 genes, followed by autoimmune diseases and non-disease traits (Figure 2D). To assess whether clusters are biologically meaningful, we performed an enrichment analysis between every cluster and hundreds of GO Biological Process (GOBP) gene sets. We theorize that significant enrichment of a cluster with a GOBP means the genes in the cluster likely function together to carry out a specific cellular process or pathway. On ConsensusPathDB, for autoimmune and complex diseases, the median proportion of GOBP enriched clusters are >0.75 and >0.60 , respectively, suggesting most clusters are biologically relevant (Figure 2E). In contrast, most clusters in non-disease traits are not enriched for a GOBP (Figure 2E).

### 3.2 Isolating CI-enriched disease clusters

Clusters of related, disease-associated genes on functional gene interaction networks are likely to correspond to the pathways and biological processes disrupted during disease progression. For complex disorders, multiple pathways are likely to be affected. Our next goal was to identify which cluster(s) within a set of disease-associated genes corresponds to the CI component of the disease. For this analysis, similar to the expansion of disease- or trait-associated genes, we used GenePlexus to predict novel inflammation genes for each of the five sets of inflammation-related seed genes procured from different sources (see Methods Section 2.5.1, Supplementary Table S4). We then scored the enrichment of CI genes in each disease cluster and performed a permutation test using 5,000 random gene sets for each disease to determine the significance of the enrichment score (see Methods Section 2.5.2 and Section 2.5.3 and Figure 1A steps 3–5, Supplementary Table S5).

**FIGURE 3:**
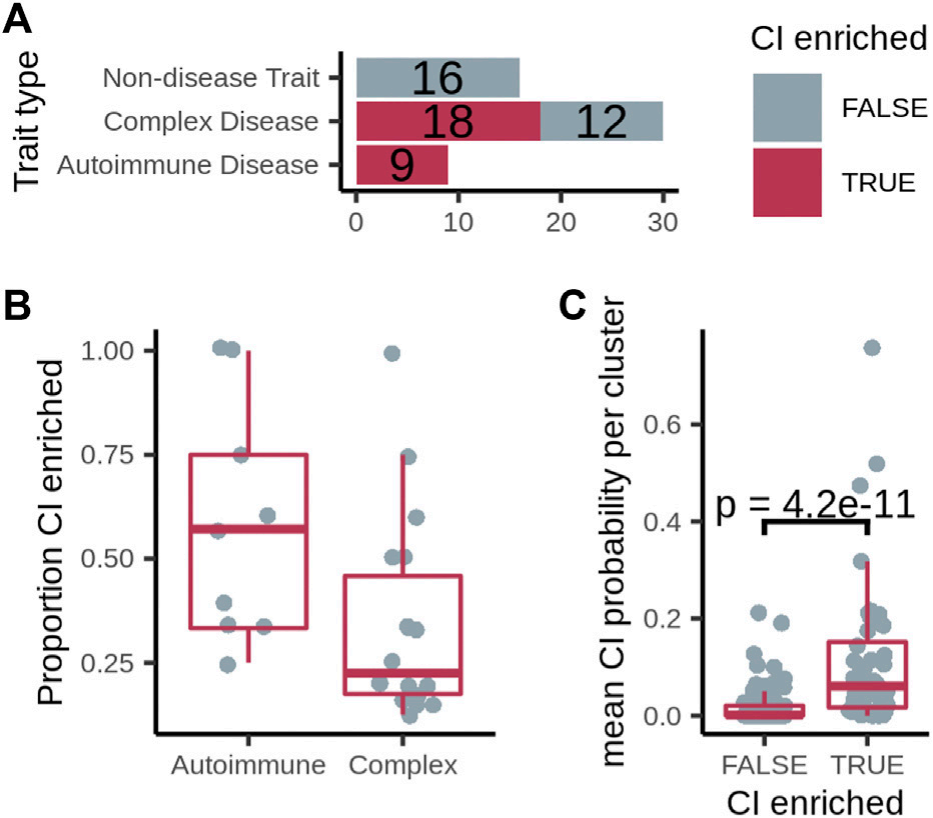
**(A)** Number of diseases/traits with at least one cluster overlapping the expanded chronic inflammation (CI) geneset (dark pink), out of the total number of diseases/traits. **(B)** The proportion of CI-enriched disease clusters among all disease clusters per disease. **(C)** Mean probability that genes with no known relationship with chronic inflammation residing in a CI-enriched cluster or non-CI-enriched cluster are associated with CI. *p*-value calculated using a one-sided Fisher’s Exact test.

With various base networks and CI gene sources, we tested all network–CI-geneset combinations and chose the one that resulted in the highest proportion of autoimmune diseases and lowest proportion of non-disease traits with at least one CI-enriched cluster. Based on this test, we picked ConsensusPathDB as the base network and “high-confidence Geneshot” as the source of CI genes (Supplementary Figure S2). We were able to identify clusters enriched for CI genes in all of the autoimmune disorders surveyed (9/9), while finding no CI-enriched clusters among the non-disease traits (Figure 3A). We identified at least one CI-enriched cluster in 18 of 30 of the complex diseases (Figure 3A). Twelve out of the 27 diseases with at least one CI-enriched cluster had two or more CI-enriched clusters, and the median proportion of CI-enriched clusters out of the total clusters is higher for autoimmune diseases than complex diseases (Figure 3B). The number of diseases with at least one CI-enriched cluster varied with different combinations of prediction network, cluster network, and inflammation gene set (Supplementary Table S6). In every case, however, the proportion of autoimmune diseases with at least one CI-enriched cluster was higher than that for non-disease traits suggesting that our method is robust to changes in base-network and inflammation gene set (Supplementary Figure S2).

We hypothesized that, through guilt-by-association, even the genes with no known relationship with chronic inflammation residing in a CI-enriched cluster should have a higher probability of being CI-associated than those in non-CI-enriched clusters. To test this hypothesis, we used GenePlexus with features from each gene-gene interaction network to calculate the probability that every gene is associated with each inflammation gene set. Then, focusing on the genes in disease clusters that were not present in the inflammation gene set, we found that the mean CI probability of these genes in CI-enriched clusters is significantly higher for CI-enriched clusters than non-enriched clusters in 24 out of 25 network/CI-gene set combinations (Supplementary Figure S3-S7), including ConsensusPathDB with the high-confidence Geneshot CI gene set (Figure 3C). This observation suggests that the CI-enriched clusters as a whole, and not just the genes in the high-confidence Geneshot CI gene set residing within them, are CI-associated in the disease of interest. Knocking out putative inflammation associated genes in animal models of the appropriate disease and testing for an increase in known inflammation markers would confirm this result.

### 3.3 Comparing CI gene signatures across diseases

**FIGURE 4:**
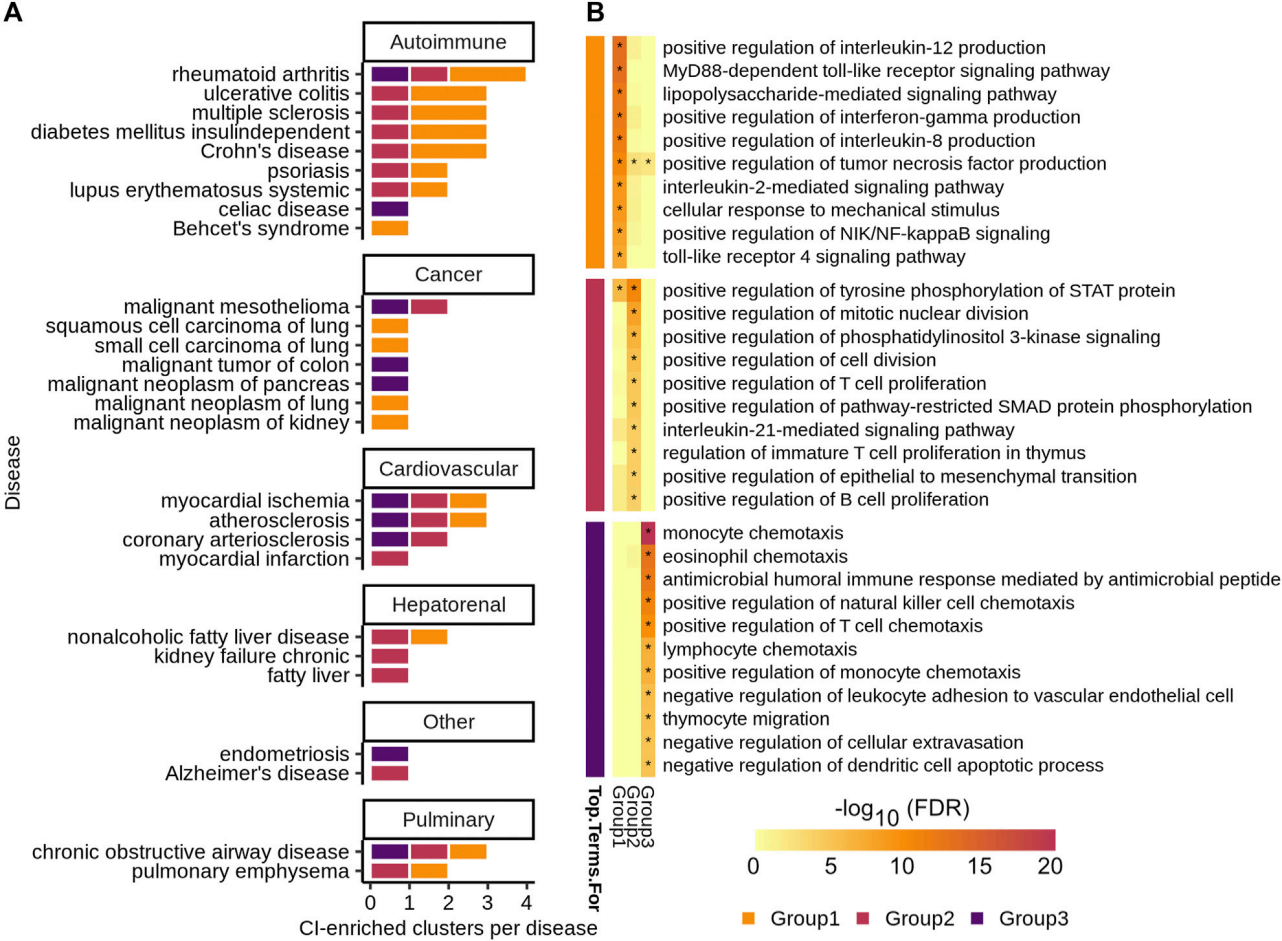
**(A)** Number of CI-enriched clusters per disease colored by CI-signature group. **(B)** Top ten enriched GOBP categories by Benjamini–Hochberg procedure corrected FDR for each CI-signature group—the group is denoted by the colored blocks to the left of the heatmap. The heatmap shows the ${-\log}_{10}\left(FDR\right)$ of the enrichment for each CI-signature group — * denotes p< 0.05 .

To determine if related diseases have similar chronic inflammation signatures, we used a network-based approach to quantify the similarity between each pair of ConsensusPathDB/high-confidence GeneShot CI-enriched disease clusters across diseases and grouped similar clusters together using the Leiden algorithm (Traag et al., 2019; Fiscon et al., 2021) (Figure 1B, steps 1–2). Several diseases have more than one CI-enriched cluster and none of these diseases have clusters belonging only to one group (Figure 4A, Supplementary Table S7). Moreover, diseases belonging to the same broad category—i.e. autoimmune, cancer, or cardiovascular disease—do not have a larger proportion of clusters belonging to a particular group than expected by chance (one-sided Fisher’s exact test, Figure 4A). This suggests that one disease can harbor more than one type of chronic-inflammation signature, and that the same signatures can be found in very different diseases. For example, rheumatoid arthritis, myocardial ischemia, atherosclerosis, and chronic obstructive airway disease all have CI-enriched clusters belonging to each of the three signature groups.

To determine the biological significance of these signature groups, we performed enrichment analyses for genes unique to each group among GO biological processes (Figure 4B, Supplementary Table S8). The top 10 significantly enriched terms for each group are largely distinct, with group 1 being enriched for immune relevant signaling pathways, group 2 for regulation of immune cell proliferation, and group 3 for regulation of immune cell chemotaxis (Figure 4B).

### 3.4 Predicting novel treatment opportunities

**FIGURE 5:**
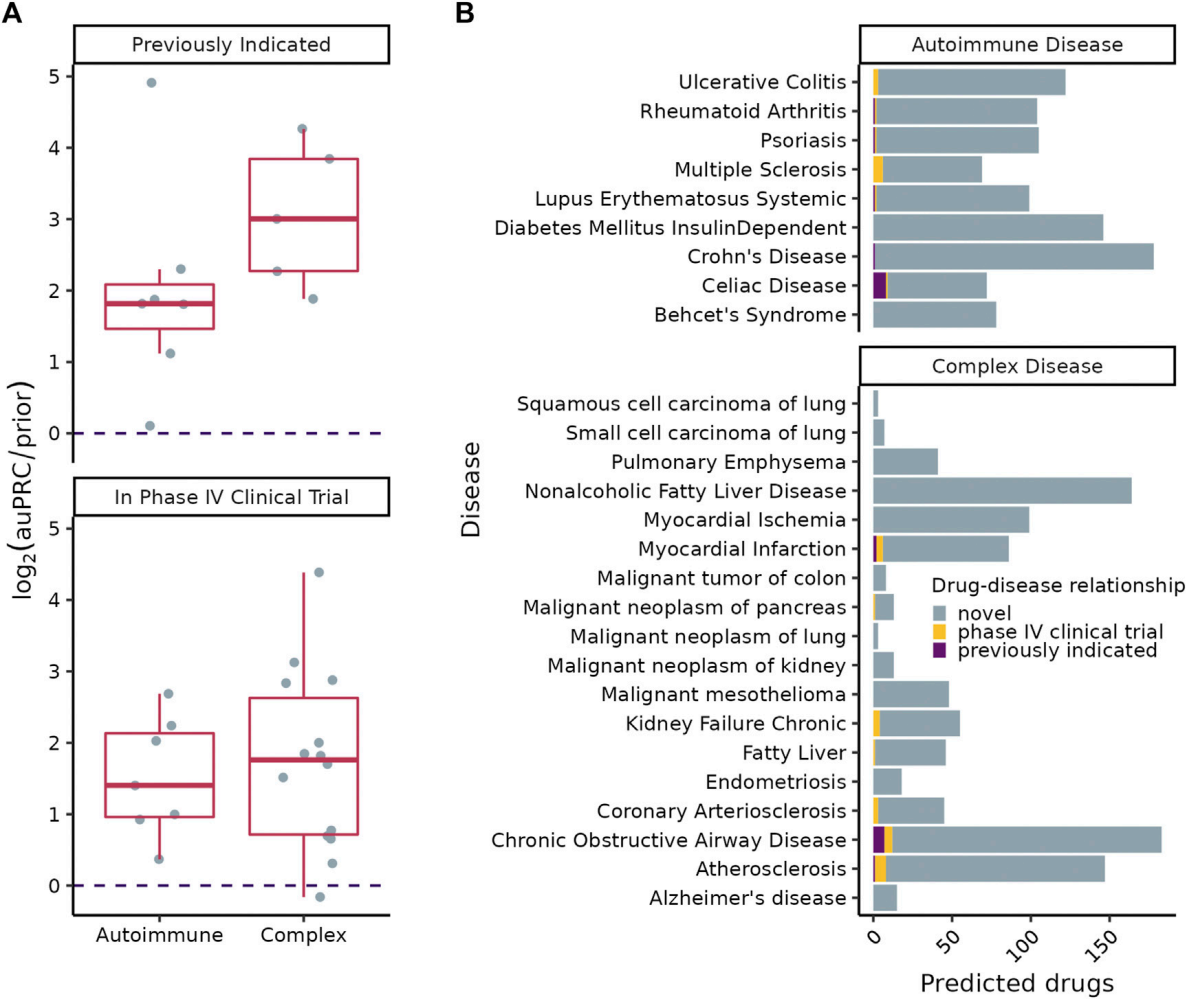
**(A)** $\log_{2}\left(auPRC/prior\right)$ of SAveRUNNER predictions using drugs previously indicated for the disease (top) or drugs ever in Phase IV clinical trials for a disease (bottom) as positive examples. The dotted line is at $\log_{2}\left(auPRC/prior\right)=0$ . $\log_{2}\left(auPRC/prior\right)>0$ denotes predictions better than random chance. **(B)** Number of SAveRUNNER predicted genes (Benjamini–Hochberg procedure corrected FDR<0.01 ) per disease.

Our final goal was to leverage the ConsensusPathDB/high confidence GeneShot CI-enriched disease clusters we discovered to find potential avenues for repurposing approved drugs to therapeutically target systemic inflammation underlying complex diseases (Figure 1B, step 3). Towards this goal, we used SAveRUNNER to find associations between CI-enriched clusters and FDA approved drugs through each drug’s target genes (Fiscon et al., 2021). We found that SAveRUNNER predictions for known treatments were better than random chance — $\log_{2}\left(auPRC/prior\right)>0$ — for diseases with at least five known treatments (Figure 5A). Moreover, with the exception of myocardial ischemia, SAveRUNNER predicted drugs in Phase IV clinical trials better than random chance (Figure 5A) (AACT Database, 2022). Drugs in Phase IV are those that have already been proved effective for treating a disease (in Phase III) and are being monitored for long-term safety and efficacy.

SAveRUNNER predicted between 3 and 178 high-confidence ( FDR< 0.01 ) treatments for each disease and identified previously indicated drugs for five of the nine autoimmune disorders (Figure 5B, Supplementary Table S9), with significant enrichment among drug predictions for celiac disease (one-sided Fisher’s exact test, BH corrected FDR< 0.001 ). SAveRUNNER found previously indicated treatments for only three of the 18 complex diseases (Figure 5B, Supplementary Table S9). This result is expected given that, unlike for autoimmune disorders, most known treatments for these complex disorders are not likely to target the immune system. Treatments previously tested in a clinical trial were predicted for six autoimmune disorders and seven of the complex disorders (Figure 5B).

**FIGURE 6:**
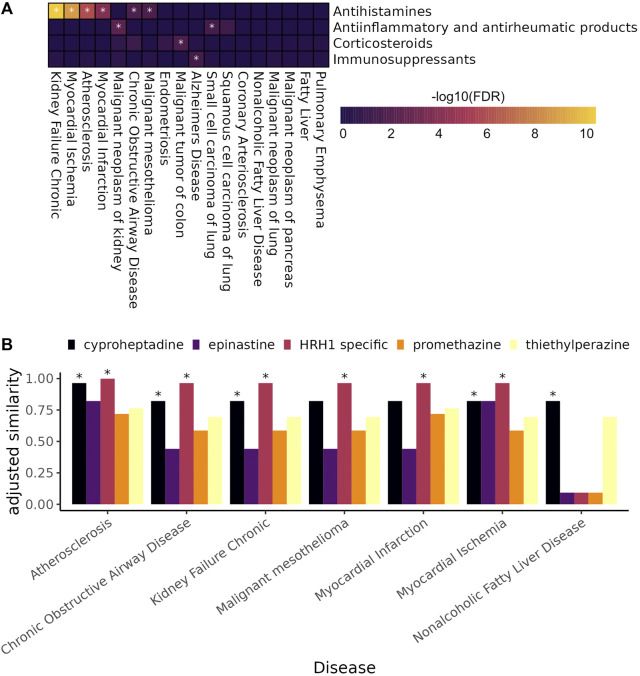
**(A)** Heat map showing the enrichment of anti-inflammatory and immunomodulating drugs among highly ranked SAveRUNNER predicted drugs (gene set enrichment analysis, * denotes *adjusted* p−value< 0.05 ). **(B)** Bar plot showing the adjusted similarity scores of antihistamines for complex diseases with at least one antihistamine among drugs predicted by SAveRUUNER to treat the disease — * denotes FDR<0.01 . HRH1 specific antihistamines are those listed in our high-confidence drug target database as only targeting *HRH1*.

We tested for enrichment of drugs belonging to four immune-related drug classes among treatment predictions highly ranked by SAveRUNNER for each complex disorder (Figure 6A). SAveRUNNER allows for drug prioritization based both on the *p*-value and on the adjusted similarity score between drug target genes and CI-enriched cluster genes. Highly scoring drug-cluster pairs have genes that are closely related in the gene interaction network, which increases the likelihood that the drug will be on-target for the paired disease (Fiscon et al., 2021). We found that antihistamines as a whole are enriched for six of the 18 complex disorders (Figure 6A). Antihistamines that specifically target histamine receptor H1 (*HRH1*) have the highest adjusted similarity score for six of the seven complex disorders with any antihistamine among their high-confidence targets (Figure 6B). SAveRUNNER predicted that cyproheptadine, which targets both *HRH1* and the serotonin 2A receptor, *HTR2A*, instead of *HRH1* alone would be the best antihistamine for treating non-alcoholic fatty liver disease (Figure 6B). While cyproheptadine is also a high-confidence predicted treatment for atherosclerosis, myocardial ischemia, and chronic obstructive airway disease, it is unlikely to be an effective treatment for myocardial infarction or malignant mesothelioma (Figure 6B). Interestingly, of the eight diseases, only myocardial infarction and malignant mesothelioma do not have a CI-enriched cluster belonging to CI-signature group 2 (Figure 4A). This finding suggests that, even among drugs in the same class, we can predict disease-specific treatments for the chronic inflammation component of the disease etiology.

## 4 Discussion

Complex diseases exhibit a staggering amount of heterogeneity, being associated with hundreds of genes and with a range of phenotypes. Therefore, to continue advancing our understanding of disease mechanisms and our ability to treat these diseases, it is critical to deconvolve disease heterogeneity by: a) resolving subsets of disease genes (and cellular processes/pathways) that underlie specific disease-associated phenotypes, and b) identifying avenues to diagnostically and/or therapeutically target those specific phenotypes.

Here, we present a computational data-driven approach to address this critical need (Figure 1). We used our approach to study chronic inflammation (CI) — a phenotype present across many complex diseases. We generated comprehensive lists of (known and predicted) disease-associated genes and identified and classified the CI signal among these genes. We used these signatures to predict novel treatment options to target the inflammatory components of 18 complex diseases.

A key aspect of our approach is ensuring its sensitivity to detect CI disease signatures using autoimmune diseases as positive controls. In autoimmune diseases, the immune system mistakenly attacks healthy tissue causing long-term systemic inflammation. Thus, we expect that the underlying CI disease signatures would be easily identifiable by a valid approach. Indeed, in each of the nine autoimmune diseases analyzed, our approach isolated gene clusters enriched for CI genes (Figure 1A), and identified drugs already used to treat a number of these disorders (Figure 5B). This finding is encouraging given that we conservatively matched drugs to diseases only based on expert-curated drug-target data from DGIDb (Freshour et al., 2021) rather than using all drug-target information in DrugCentral (Avram et al., 2021).

To show that our method was not erroneously uncovering CI signals where there were none, we identified UK Biobank traits not patently associated with CI (along with their genes) to use as negative controls. Following this analysis, we found that the median fraction of trait-associated genes predicted by GenePlexus and the median fraction of genes assigned to sizable clusters were lower for these traits than for autoimmune and complex diseases (Figures 2C,D). Given that GenePlexus is a method that leverages network connectivity for predicting new genes belonging to a set, these results suggest that the genes associated with non-disease traits may not be as highly connected to one another in ConsensusPathDB as the autoimmune and complex disease genes. Moreover, most of the non-disease trait clusters were not enriched with genes annotated to GO biological processes, suggesting that these clusters are diffuse and that the member genes are unlikely to work together to support a coherent biological task. While non-disease traits like coffee intake and handedness have been associated with inflammation (Searleman and Fugagli 1987; Paiva et al., 2019), this analysis (using GWAS-based trait-associated genes) suggests it is unlikely that SNPs in a coordinated inflammation pathway influence non-disease traits and more likely that any association with inflammation is environmental, not genetic. Taken together, these results suggest that these chosen traits serve as reasonable negative controls and offer a way to meaningfully contrast the results from complex diseases. Ideally, diseases or traits with no underlying inflammatory component but with associated genes that cluster in a network (as well as the autoimmune and complex disease) will serve as better negative controls. Given how common inflammatory processes are in disease, however, such diseases are difficult to definitively identify.

Complex disorders like cardiovascular diseases, diabetes, cancer, and Alzheimer’s disease are among the leading causes of death and disability among adults over 50 years of age, and all are associated with underlying systemic inflammation (Furman et al., 2019; Vos et al., 2020). Patients with systemic inflammation caused by autoimmune disorders are more likely to have another CI disorder like cardiovascular disease, type 2 diabetes mellitus, and certain types of cancer (Armstrong et al., 2013; Dregan et al., 2014; Yashiro 2014). Further, treating one chronic-inflammatory disease can reduce the risk of contracting another, suggesting a common underlying pathway (Fullerton and Gilroy 2016). For example, treating rheumatoid arthritis with tumor necrosis factor (TNF) antagonists lowers the incidence of Alzheimer’s disease and type II diabetes (Antohe et al., 2012; Chou et al., 2016).

To better understand how CI-associated disorders relate to one another, we used a network-based approach to quantify the similarity between their CI-enriched clusters. We hypothesized, for example, that Crohn’s disease and “malignant tumor of colon” would have similar CI-signatures, given that patients with inflammatory bowel disease are at increased risk for developing colorectal cancer (Shah and Itzkowitz 2022). However, Crohn’s disease CI-enriched clusters are members of signature groups 1 and 2, while the “malignant tumor of colon” CI-enriched cluster belongs to group 3 (Figure 4A). Instead of sharing CI-signatures, related CI diseases may, instead, have complementary signatures. Indeed, the group 1 signature, which characterizes two of the three Crohn’s disease CI-enriched clusters, is enriched for genes that positively regulate proinflammatory cytokines TNF and in interferon-gamma (IFNɣ) (Figure 4B). When these cytokines bind to their respective receptors, reactive oxygen species are generated causing oxidative stress (Chatterjee 2016). Oxidative stress, in turn, induces DNA-damage that can lead to tumor formation. Colorectal tumors are infiltrated with lymphocytes, which mediate the recruitment of immune cells that suppress tumor growth (Idos et al., 2020). Immune cell infiltration likely leads to our ability to detect the group 3 CI-signature among genes associated with “malignant tumor of colon”, given that group 3 is enriched for immune cell migration and chemotaxis (Figures 4A,B). Alternatively, there is a possibility that every CI-associated disease actually exhibits all three CI-signatures, and our method is only sensitive enough to detect these in a handful of diseases.

Common treatments for systemic inflammation, including non-steroidal anti-inflammatory drugs (NSAIDs), corticosteroids, and biologics like TNF antagonists, can cause adverse effects when used long term. For instance, patients treated with corticosteroids or TNF antagonists have increased risk of infection (Rosenblum and Howard 2011; Murdaca et al., 2015; Shah and Itzkowitz 2022), and corticosteroid use increases both the risk of fracture (Kanis et al., 2004; Mitra 2011) and the risk of developing type II diabetes (Blackburn et al., 2002). NSAIDs present a unique set of side effects, particularly in elderly patients, including gastrointestinal problems ranging from indigestion to gastric bleeding, and kidney damage (Griffin 1998; Griffin et al., 2000; Marcum and Hanlon 2010). Therefore, the search for better treatment options for CI is ongoing.

Here, we leverage the CI-signatures to identify novel treatment opportunities for the CI-component of 18 complex diseases (Figure 5B). Interestingly, antihistamines were among the top drug associations for six of 18 complex diseases (Figure 6A), including atherosclerosis. Atherosclerosis is characterized by the deposition of cholesterol plaques on the inner artery walls. Mast cells, immune cells best known for their response to allergens, are recruited to arteries during plaque progression, where they release histamines. Histamines then activate the histamine H_1_-receptor, increasing vascular permeability, which allows cholesterol easier access to arteries promoting plaque buildup (Rozenberg et al., 2010). Mepyramine, one of the HRH1-specific antihistamines highly associated with atherosclerosis, has already been shown to decrease the formation of atherogenic plaques in a mouse model of the disease (Rozenberg et al., 2010). Interestingly, it is not predicted as a treatment for myocardial ischemia, which occurs when plaque buildup obstructs blood flow to a coronary artery, suggesting disease-specific antihistamine efficacy even among related diseases. Cetirizine and fexofenadine are also HRH1-specific antihistamines highly associated with atherosclerosis but neither prevented or reduced atherosclerosis progression in a mouse model of atherosclerosis, and both increased atherosclerotic lesions at low doses (Raveendran et al., 2014). In the expert-curated drug-target database used in this study, the histamine H_1_-receptor is the only target listed for all three drugs; however, the contradictory results from Rosenberg *et al.* and Raveendran *et al.* suggests that drug-specific off-target effects are mediating atherosclerosis treatment outcomes. A more complete understanding of drug-gene targets would allow for better predictions of novel disease treatments.

For example, unlike the other diseases with antihistamines as predicted treatments, only cyproheptadine, and not the HRH1-specific drugs, is likely to be an effective treatment for non-alcoholic fatty liver disease (NAFLD) (Figure 6B). Cyproheptadine is an antagonist for both histamine receptor H1 and the serotonin 2A receptor (*HTR2A*), suggesting that blocking the serotonin 2A receptor could be specifically helpful for ameliorating symptoms of NAFLD. Indeed, liver-specific *Htr2a* knockout mice are resistant to high-fat diet induced hepatic steatosis and increased fat in the liver (Choi et al., 2018). Moreover, increased serum serotonin levels were correlated with increased disease severity in patients with NAFLD (Wang and Fan. 2020).

Overall, we have shown that our method is capable of isolating the chronic inflammation gene signature of a complex disease using a network-based strategy and, by integrating information across multiple complementary sources of data, it can predict and prioritize potential therapies for the systemic inflammation involved in that specific disease. Importantly, our approach provides a blueprint for identifying and prioritizing therapeutic opportunities for any disease endophenotype.

## Data Availability

The code and data used to generate the results can be found on github repository https://github.com/krishnanlab/chronic-inflammation and Zenodo record https://zenodo.org/record/6858073 (doi: 10.5281/zenodo.6858073), respectively.
