# Supplementary figures and images for: A network-based approach for isolating the chronic inflammation gene signatures underlying complex diseases towards finding new treatment opportunities
Source: Front Pharmacol. 2022 Oct 12;13:995459. doi: 10.3389/fphar.2022.995459 (PMC9597699; doi:10.3389/fphar.2022.995459)

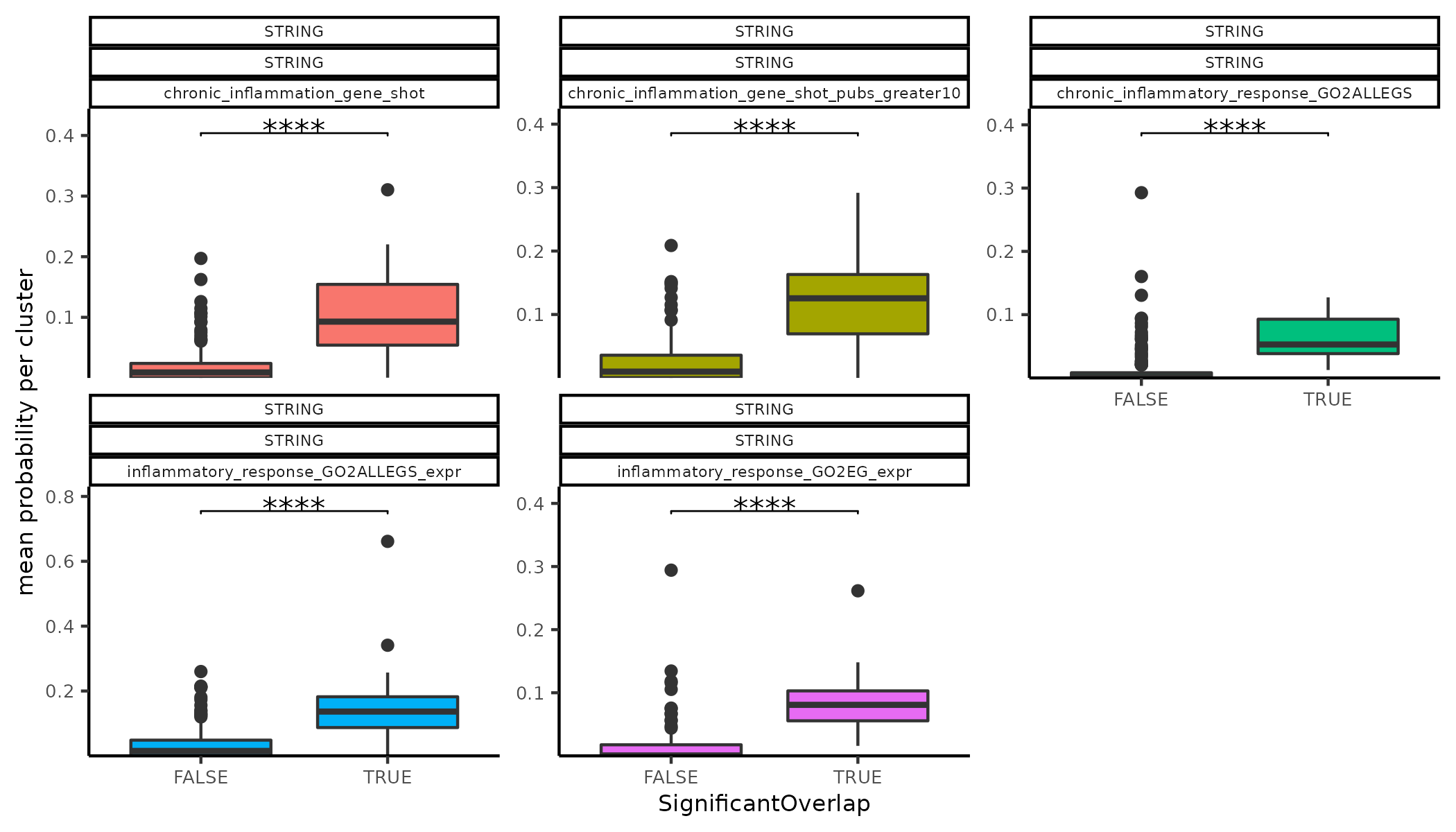

Supplement: Supplementary file 5 [file Image5.PNG]

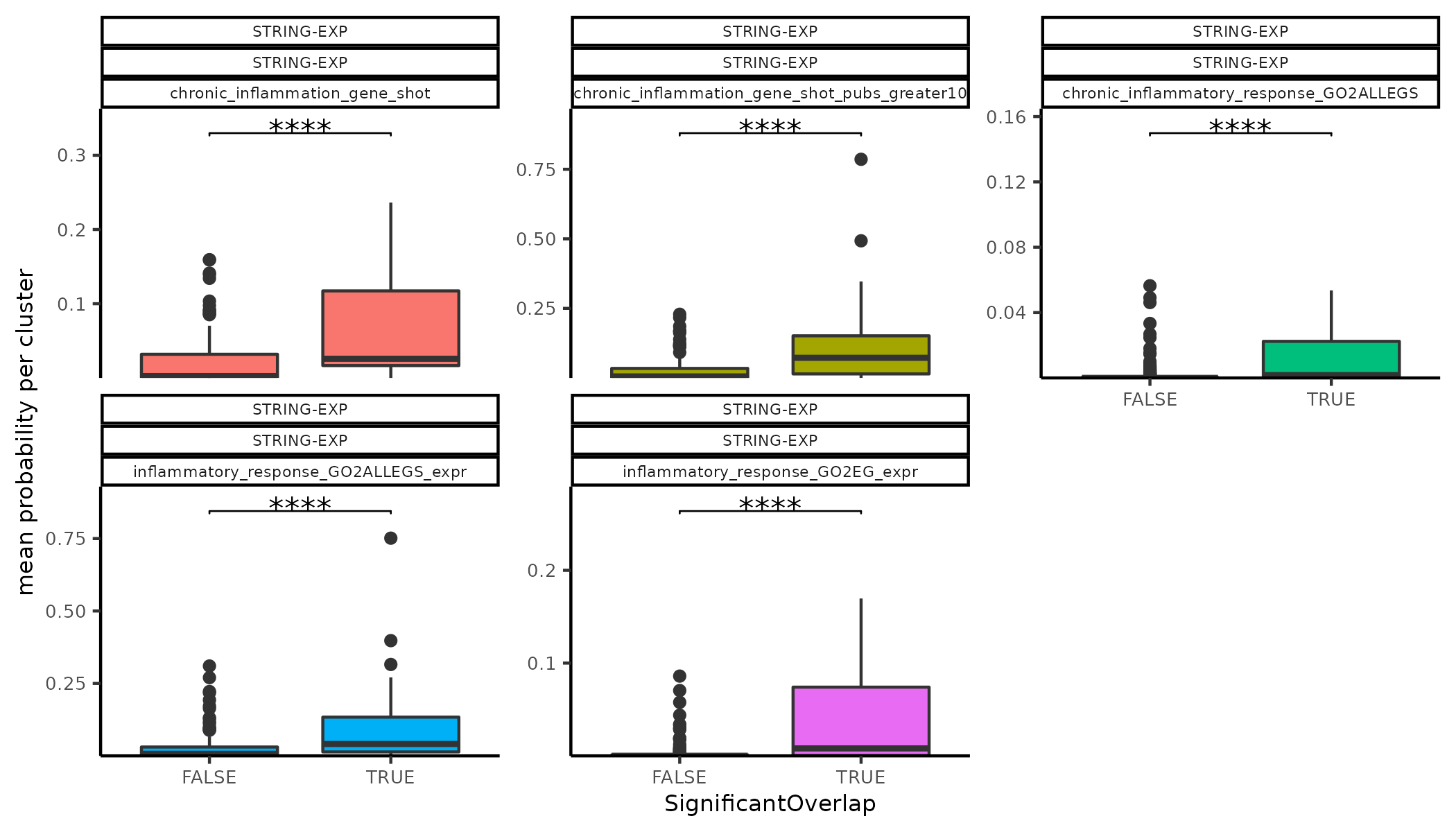

Supplement: Supplementary file 6 [file Image4.PNG]

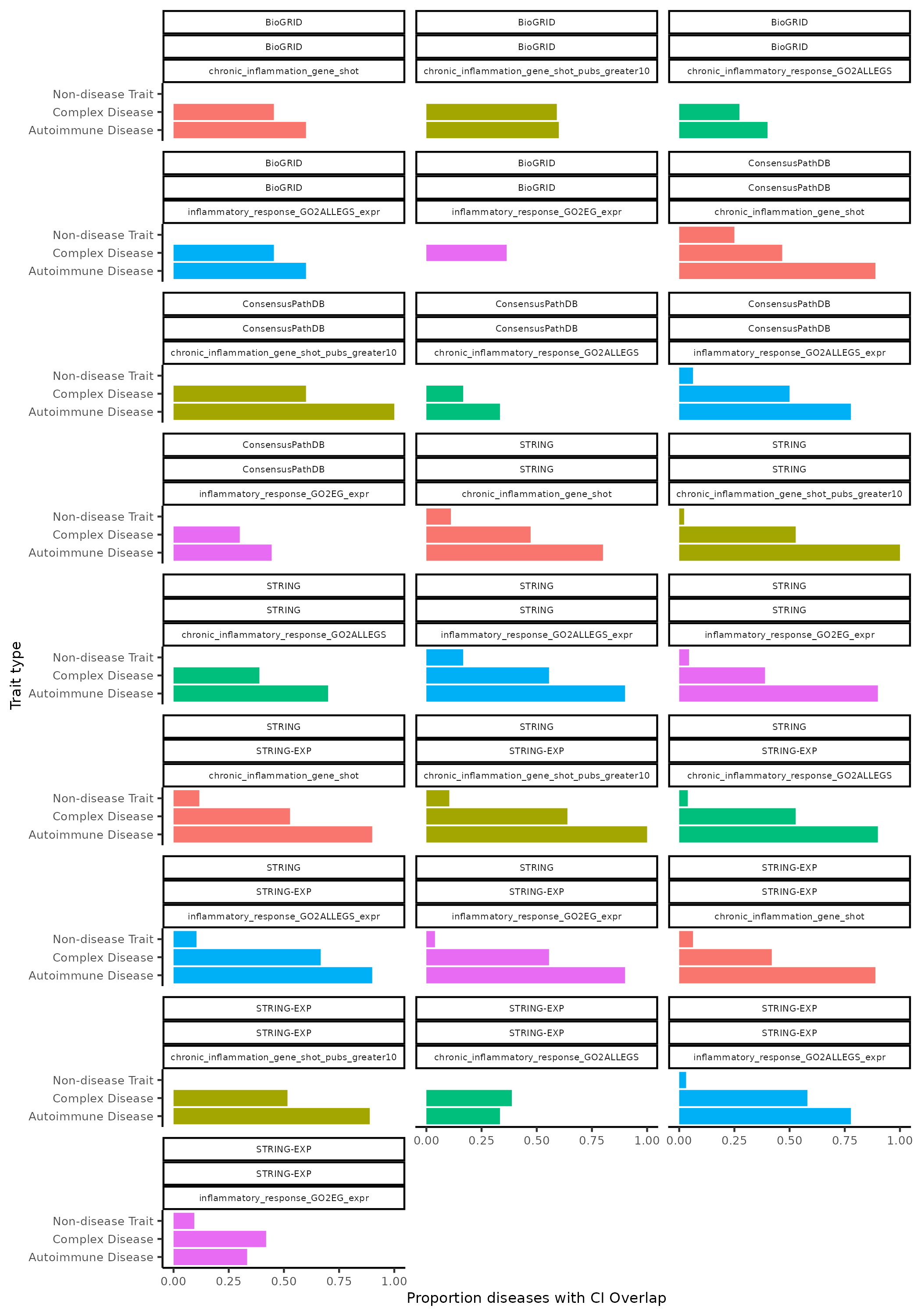

Supplement: Supplementary file 8 [file Image7.PNG]

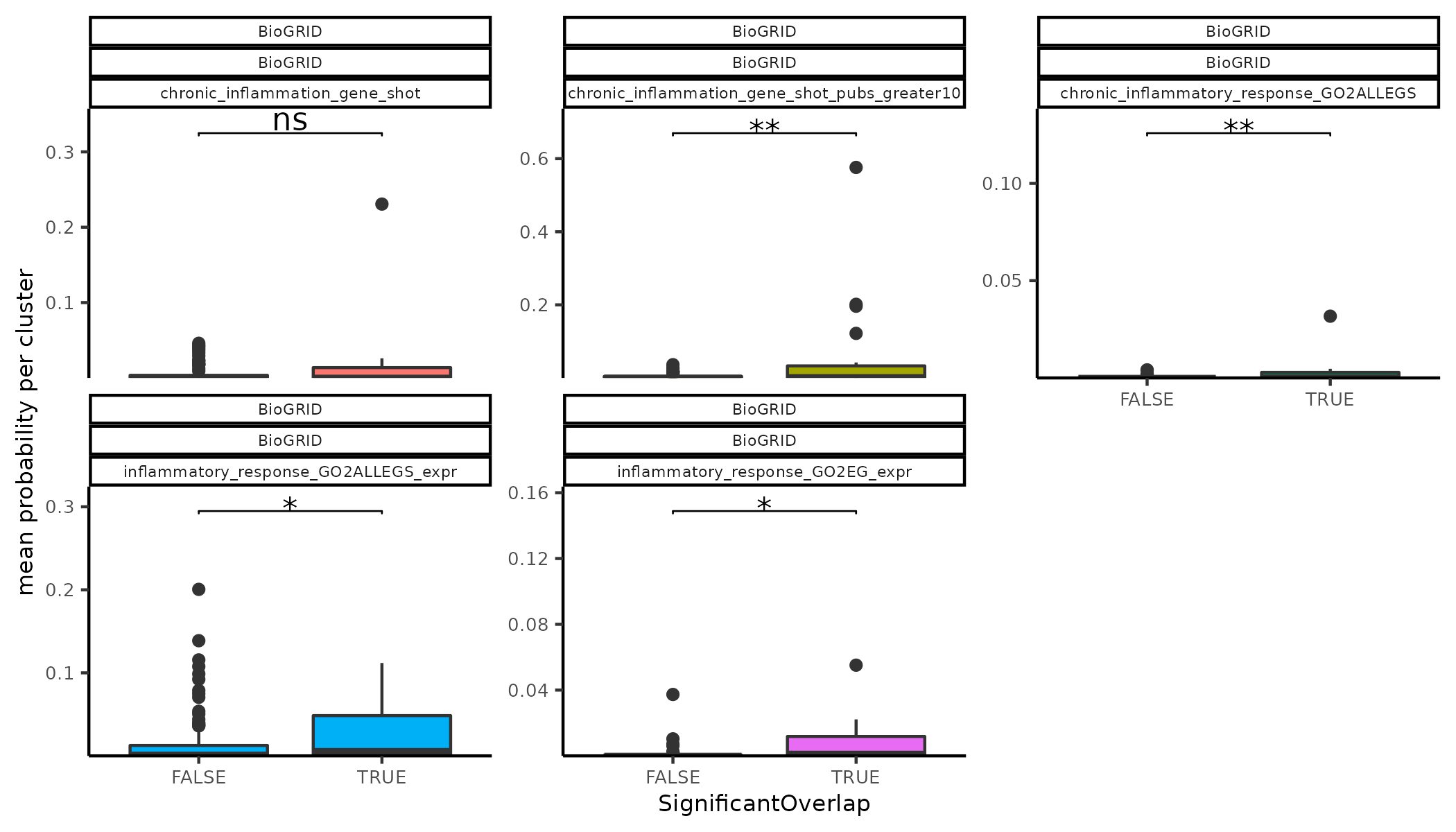

Supplement: Supplementary file 9 [file Image2.PNG]

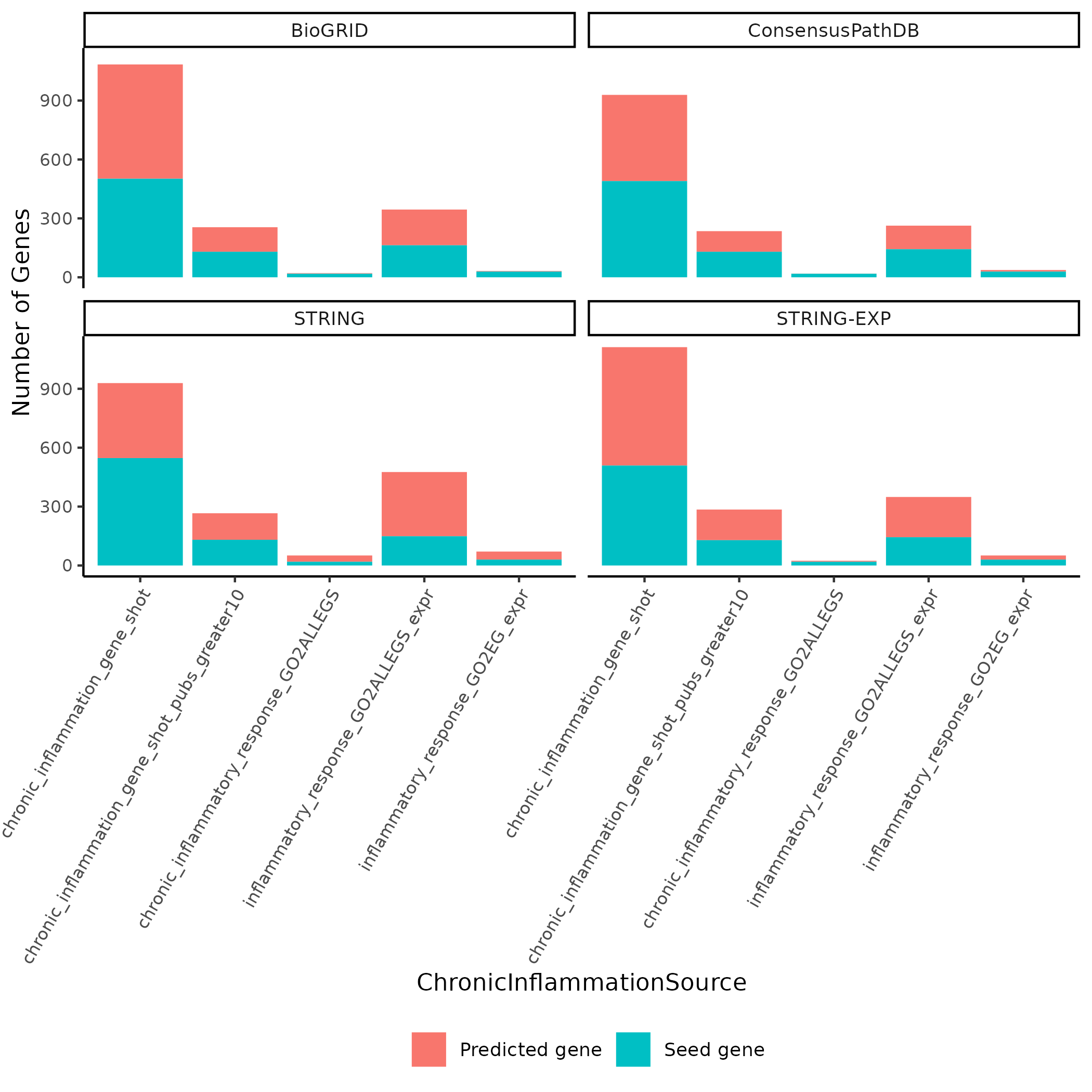

Supplement: Supplementary file 12 [file Image1.PNG]

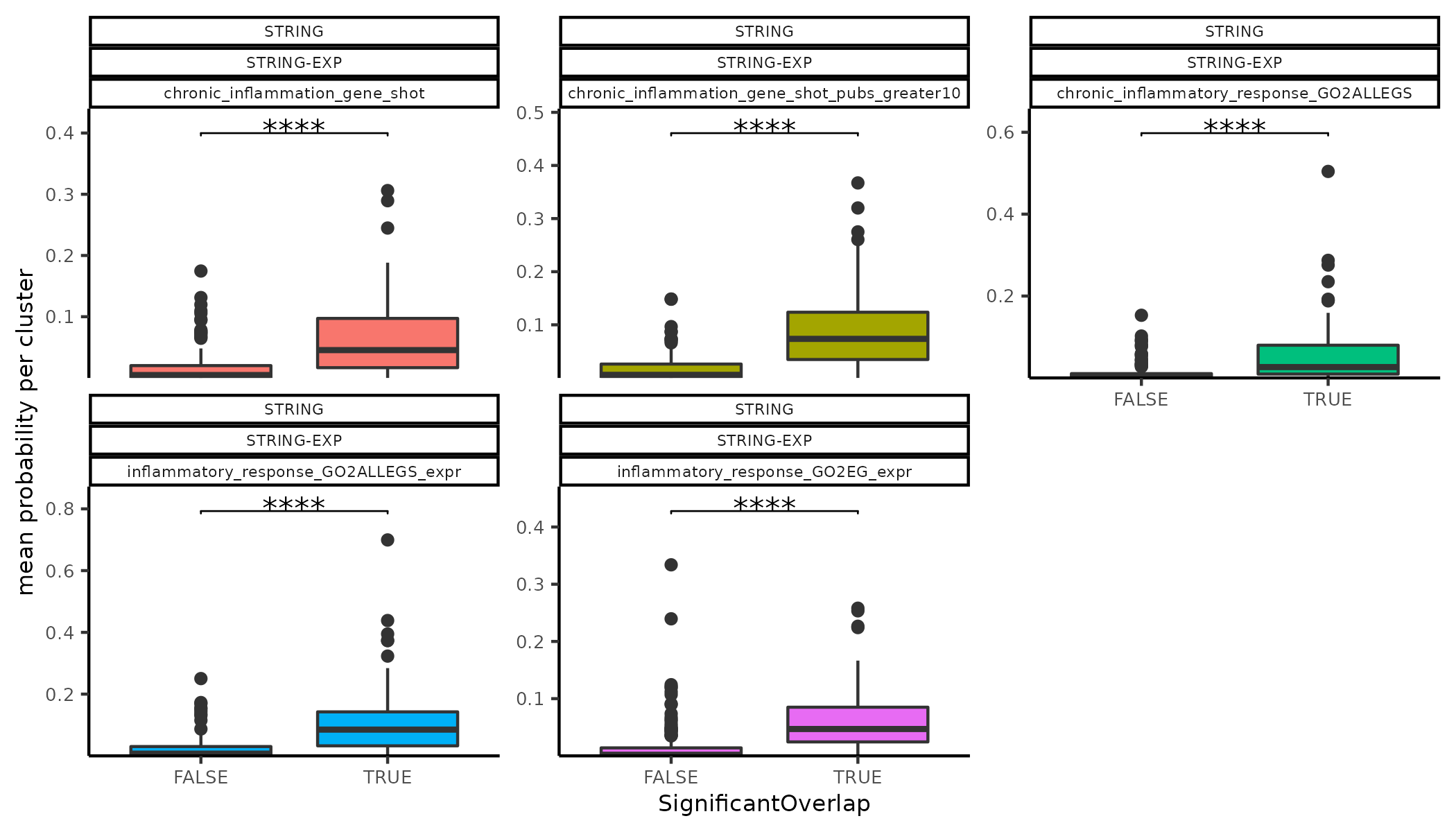

Supplement: Supplementary file 13 [file Image6.PNG]

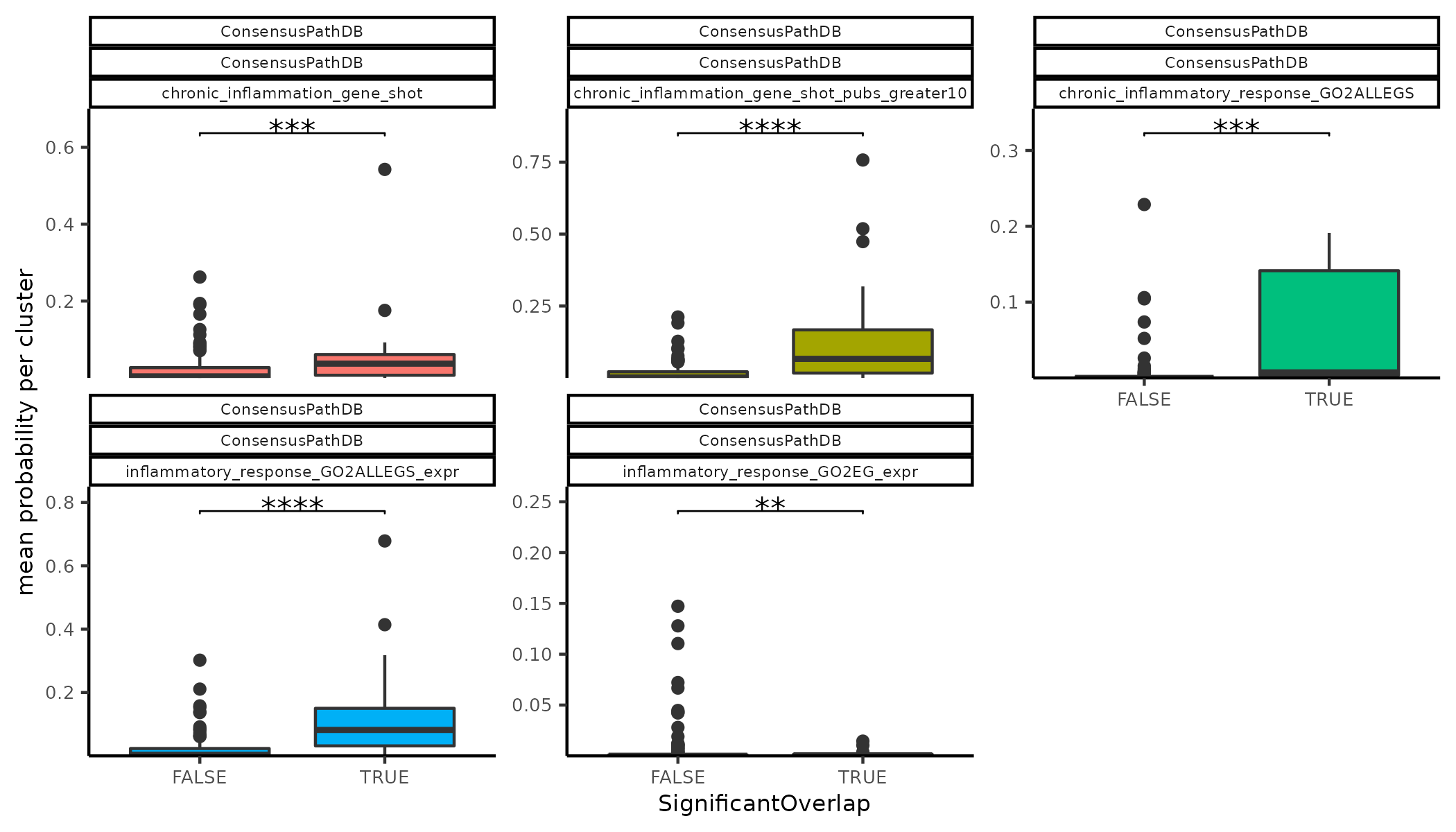

Supplement: Supplementary file 15 [file Image3.PNG]
